# Toll-Like Receptors in Acute Kidney Injury

**DOI:** 10.3390/ijms22020816

**Published:** 2021-01-15

**Authors:** Cristina Vázquez-Carballo, Melania Guerrero-Hue, Cristina García-Caballero, Sandra Rayego-Mateos, Lucas Opazo-Ríos, José Luis Morgado-Pascual, Carmen Herencia-Bellido, Mercedes Vallejo-Mudarra, Isabel Cortegano, María Luisa Gaspar, Belén de Andrés, Jesús Egido, Juan Antonio Moreno

**Affiliations:** 1 Renal, Vascular and Diabetes Research Laboratory, IIS-Fundación Jiménez Díaz, Universidad Autónoma de Madrid, 28040 Madrid, Spain; cvazqu01@ucm.es (C.V.-C.); srayego@quironsalud.es (S.R.-M.); lucas.opazo@quironsalud.es (L.O.-R.); carmen.herencia@quironsalud.es (C.H.-B.); 2 Maimonides Biomedical Research Institute of Cordoba (IMIBIC), Hospital Universitario Reina Sofía, 14004 Córdoba, Spain; Melania.guerrero@imibic.org (M.G.-H.); Cristina.garcia@imibic.org (C.G.-C.); Jose.morgado@fjd.es (J.L.M.-P.); b52vamum@uco.es (M.V.-M.); 3 Spanish Biomedical Research Centre in Diabetes and Associated Metabolic Disorders (CIBERDEM), 28040 Madrid, Spain; 4 Immunobiology Department, Carlos III Health Institute, 28220 Majadahonda (Madrid), Spain; icortegano@isciii.es (I.C.); mlgaspar@isciii.es (M.L.G.); bdandres@isciii.es (B.d.A.); 5 Biomedical Research Networking Center on Cardiovascular Diseases (CIBERCV), 28029 Madrid, Spain; 6 Department of Cell Biology, Physiology and Immunology, University of Cordoba, 140471 Cordoba, Spain

**Keywords:** toll-like receptors, inflammation, acute kidney injury, drugs, therapy

## Abstract

Acute kidney injury (AKI) is an important health problem, affecting 13.3 million individuals/year. It is associated with increased mortality, mainly in low- and middle-income countries, where renal replacement therapy is limited. Moreover, survivors show adverse long-term outcomes, including increased risk of developing recurrent AKI bouts, cardiovascular events, and chronic kidney disease. However, there are no specific treatments to decrease the adverse consequences of AKI. Epidemiological and preclinical studies show the pathological role of inflammation in AKI, not only at the acute phase but also in the progression to chronic kidney disease. Toll-like receptors (TLRs) are key regulators of the inflammatory response and have been associated to many cellular processes activated during AKI. For that reason, a number of anti-inflammatory agents targeting TLRs have been analyzed in preclinical studies to decrease renal damage during AKI. In this review, we updated recent knowledge about the role of TLRs, mainly TLR4, in the initiation and development of AKI as well as novel compounds targeting these molecules to diminish kidney injury associated to this pathological condition.

## 1. Introduction

Acute kidney injury (AKI) is characterized by an acute loss of renal function. In clinical practice, AKI is defined by an elevation of creatinine plasma concentration above ≥0.3 mg/dL in the first 48 h, an urine volume below 0.5 mL/kg/h for 6 h, or an 1.5 fold increase in serum creatinine as compared with the baseline values [1]. Reduction in urinary volume and urinary solute excretion leads to accumulation of waste products in the body as well as dysregulation of blood pH and osmolarity, that may result lethal for the patient. Depending on the intensity of AKI, the use of dialysis for patient survival may be necessary.

In the last years, the incidence of AKI has increased considerably as a consequence of the high prevalence of AKI- associated comorbidities, such as aging, chronic kidney disease (CKD), diabetes and hypertension, among others [2]. In fact, it has been estimated that around 13.3 million people/year develop AKI [3]. 

Many people fully recover renal function after the AKI episode, however there are patients that progress to CKD, suggesting adverse chronic outcomes [4]. Indeed, AKI patients have a higher risk to develop CKD than healthy individuals. Moreover, AKI is associated with high frequency of cardiovascular events and both early and long-term mortality [5]. Despite these adverse outcomes, there are no specific treatments to reduce chronic renal damage after AKI. Therefore, it is necessary a better comprehension of the physiopathology associated to this syndrome to identify novel therapeutic approaches. Toll like receptors (TLRs) are immunity sensors that recognize a wide variety of endogenous and exogenous molecules present in AKI and promote activation of intracellular pathways associated to renal damage [6]. In this review, we updated recent knowledge about the role of TLRs as potential drug targets to prevent and/or retard AKI-associated complications.

## 2. Pathophysiology of AKI

The etiology and pathophysiology of AKI are complex and multifactorial. AKI can be classified into three different types: pre-renal, intrinsic and post-renal. Pre-renal AKI is associated to a decreased renal blood flow by hypovolemia, impaired cardiac function, systemic vasodilation or increased vascular resistance, thus leading to a reduced glomerular filtration rate (GFR). Intrinsic AKI is related to direct injury or nephrotoxicity of parenchymal renal cells (glomeruli, tubules, interstitium and endothelial cells). Post-renal AKI is mainly related to a reduction in GFR as consequence of increased intra-tubular pressure by obstruction of urinary tract [7]. 

The underlying pathophysiological mechanisms in AKI include hemodynamic changes, direct tubular toxicity (mainly in proximal tubular cells), obstruction and dysfunction of microvascular vessels, congestion of tubular lumen, and renal inflammation [8]. These pathogenic mechanisms may co-exist in AKI-patients, thus complicating diagnosis and treatment. 

AKI depends on the duration and severity of the insult [9]. When acute renal damage occurs, there is a first phase of tubular death, followed by a phase of cell regeneration and recovery of the renal function. Massive tubular cell death results from different causes, such as toxic insults, sepsis, oxidative stress, or ischemia, among others [10]. If the cause of kidney damage is prolonged over time, it can trigger more severe tubular cell death. During this process, tubular cells release chemokines, cytokines and other inflammatory stimuli that promote leucocyte infiltration in the kidney [11,12]. Inflammation is important for the regeneration and replacement of necrotic cells during AKI [13,14]. However, exacerbated or unresolved inflammation triggers the activation of fibrosis, a phenomenon that may be involved in progression to CKD after AKI [8].

## 3. Biomarkers in AKI

In the current clinical practice, AKI diagnosis is mostly based in determination of serum creatinine concentration [15]. However, the levels of this nitrogen-containing compound only increase when kidney injury is well stablished, restricting the possibility to detect early phases of AKI [16]. In addition, many factors influence serum creatinine concentration e.g., age, gender, diet, muscle mass, and hydration status), limiting its utility as an AKI biomarker. For these reasons, there is a great interest in the search of new AKI biomarkers for early detection, differential diagnosis and prognosis [17,18]. In this context, the most promising AKI biomarkers are listed in Table 1. These novel biomarkers are related with pathological processes involved in AKI development, such as inflammation, oxidative stress and renal cells death [19,20]. Furthermore, current studies support the potential value of circulating and urinary miRNAs as novel AKI biomarkers e.g., miR-21, miR-30a-e and miR-494, among others [18,21,22].

## 4. Toll-Like Receptors: Structure, Localization and Function

TLRs are composed by 10 members in humans (TLRs 1–10) and 12 in mice (TLRs 1–9, and TLRs 11–13) [23]. TLRs are type I transmembrane glycoproteins with a structure composed of three regions: (1) a *N*-amino terminal extracellular domain containing leucine-rich motifs with a horseshoe-like structure responsible for ligand recognition, (2) a transmembrane domain and (3) a conserved cytoplasmic TIR (toll-interleukin 1 receptor) domain, required for the activation of downstream signal pathways [24]. 

TLRs recognize PAMPs (pathogen-associated molecular patterns), which are structural motifs found in viruses, fungi, and bacteria [25]. Cell membrane TLRs (TLR4, TLR5 and TLR10 as well as heterodimers of TLR2 with TLR1 and TLR6) are recruited to phagosomes after activation by their respective ligands e.g., LPS, lipoproteins, flagellin) [26]. By contrast, TLRs involved in the recognition of nucleic acid-like structures e.g., dsRNA, ssRNA, unmethylated CpG motifs) are localized in the endoplasmic reticulum, endosomes and lysosomes (TLR3, TLR7 and TLR9) [27]. Furthermore, TLRs can also recognize endogenous stress signal or DAMPs (damage-associated molecular patterns), including heat shock proteins (Hsp), extracellular matrix components (fibrinogen, hyaluronic acid), nuclear cytosolic proteins (HMGB1, high mobility group box protein 1) and elements from damaged cells or organelles [28]. TLRs are activated by interaction with their ligands, leading to the production of inflammatory cytokines, chemokines and interferons (IFNs). Moreover, TLRs participate in maturation and differentiation of antigen-presenting cells, thus linking innate and adaptive immune response [29]. TLRs gene expression has been reported on immune (e.g., spleen, thymus, lymph nodes) [25] and non-immune tissues (skeletal muscle, brain, heart, liver, kidneys, lung, intestine and pancreas, among others) [29], as well as all innate [30] and adaptive immune cells [31]. 

TLR4 is the best characterized TLR in AKI. Therefore, in this review we will focus on this molecule in relation to AKI progression and associated complications. TLR4 is necessary for recognizing and facilitating responses to LPS in mammals [23]. In addition to LPS, numerous TLR4 ligands have been identified, including both PAMPs (various viral fusion and envelope proteins) and DAMPs (HMGB1, Hsp60 and Hsp70, fibrinogen, fibronectin, hyaluronic acid and heme, among others) [29,32,33]. With an almost identical expression profile in humans and mice, TLR4 is mainly found in peripheral blood lymphocytes and spleen. As compared with spleen, moderate levels of TLR4 expression have been reported in lungs, intestine, ovary, and placenta, while the lowest TLR4 expression has been observed in heart, brain, liver, kidneys, muscle, pancreas, testis and thymus [34]. 

Under physiological conditions, TLR4 is widely expressed in renal parenchymal cells and resident immune cells. TLR4 expression is elevated in renal cortex, whereas low levels have been described in renal medulla. In the cortex, TLR4 expression is mainly detected in proximal and distal tubules, but also in podocytes, glomerular mesangial cells, peritubular endothelial cells and collecting duct cells [35]. In normal conditions, renal TLR4 expression is low, however the expression of this molecule increases in response to renal injury and/or infection. For example, after ischemia/reperfusion injury, increased level of TLR4 expression has been detected in renal endothelial cells [36], tubules and infiltrating leukocytes [37,38]. In sepsis-induced AKI, elevation of renal TLR4 was reported in proximal and distal tubules and in peritubular and glomerular capillaries [39]. TLR4 expression is also upregulated in the kidney in cisplatin-mediated AKI [40], cyclosporin nephrotoxicity [41], lupus nephritis [42], unilateral ureter obstruction [43], diabetic nephropathy [44] and rhabdomyolysis-induced AKI [45]. These observations suggest that TLR4 plays an important role in pathophysiology of AKI and may be a potential therapeutic target to diminish renal damage in response to these pathological stimuli. 

## 5. TLR4 Signaling Pathways

Recognition and binding of TLR4 with its respective ligands seems to require the presence of cell surface adaptors. For example, LPS binds to LBP (LPS-binding protein) and this complex is further recognized by the membrane protein CD14 [46]. CD14 transfers LPS to MD-2, a beta-cup folded protein necessary for LPS-mediated TLR4 dimerization. Therefore, TLR4 recognition and activation by LPS requires coordination of a complex consisting of LBP, CD14 and MD-2, which act sequentially to promote TLR4 signaling [47]. Diverse ligands activate TLR4 through the binding with one or more members of the LPS-multi-receptor complex [48]. However, further research is needed to identify which proteins are involved in TLR4-mediated recognition of each specific ligand. 

**Figure 1:**
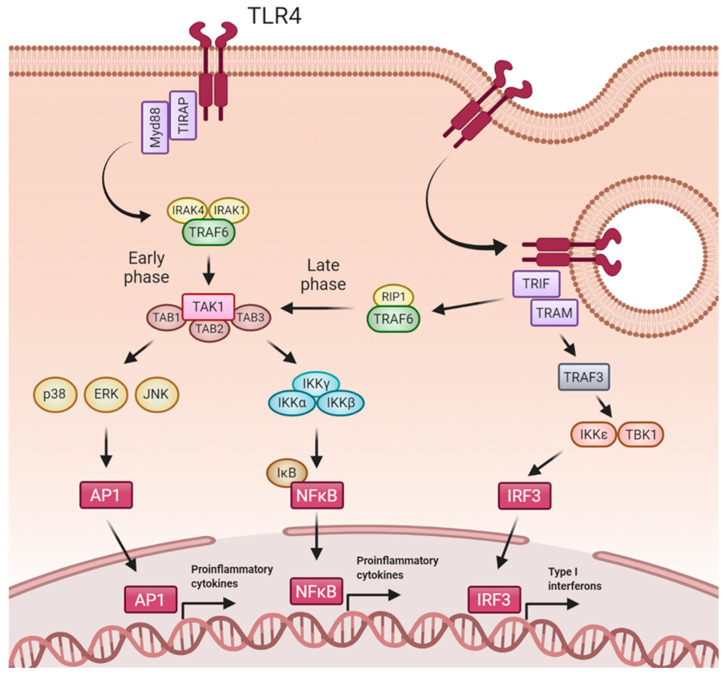
TLR4 signaling pathway. Upon ligand recognition, TLR4 forms homodimers and signals through MyD88-dependent and MyD88-independent pathways. In early phase of TLR4 activation, MyD88-dependent pathways begins with TIRAP and MyD88 recruitment to TLR4 and subsequently assembly with TRAF6, IRAK1 and IRAK4. TRAF6 associates with the complex formed by TAB1, TAB2, TAB3 and TAK1. Once activated, TAK1 mediates the phosphorylation of the IKK complex (IKKα, IKKβ, and IKKγ), which phosphorylates the inhibitory subunit IkB, resulting in nuclear NFκB translocation and proinflammatory genes expression. Besides activating NFκB, TAK1 also phosphorylates and activates MAP kinases (ERK, Jnk, p38) to further reinforce the proinflammatory cytokines expression. MyD88-independent pathway requires the recruitment of TRIF and TRAM to TLR4 after its internalization in endosomes. The subsequent association with RIP1 and TRAF6 converges in the activation pathway of NFκB in a late phase of TLR4 activation. TRAF3 activates IRF3 through IKKε and TBK1, inducing transcription of type I interferons and IFN-inducible genes. Created with BioRender.com.

In addition to LPS, TLR4 recognizes several exogenous and endogenous ligands. Upon ligand binding, TLR4 homodimerizes and initiates intracellular signaling through two major downstream pathways: (1) from the plasma membrane, the MyD88-dependent pathway, which activates early NFκB activation and cytokines production, and (2) from the endosome, the MyD88-independent TRIF-dependent pathway, which upregulates type I IFNs and a late phase NFκB activation [24,49] (Figure 1). Early phase of TLR4 activation is triggered from the cell surface after ligand recognition. TLR4-mediated MyD88-dependent signaling pathway requires the initial interaction with the sorting adaptor TIRAP (TIR domain-containing adapter protein), present in regions enriched with phosphatidylinositol 4,5-bisphosphate, such as lipid rafts [48,50]. TIRAP contains a TIR domain that enables the interaction of MyD88 with the TIR domain of TLR4, leading to the creation of a complex: the myddosome [51,52]. Thus, myddosome is composed by MyD88, TIRAP, and IRAK (interleukin-1-receptor-associated kinase). The binding of MyD88 to IRAK4 induces phosphorylation of IRAK1, which induces its kinase activity [53,54]. Then, activated IRAK1 autophosphorylates and this hyperphosphorylation enables TRAF6 (TNF-receptor-associated factor 6) to bind to this complex. IRAK-TRAF6 then interacts with a complex formed by TAK1 (TGF-β Activated Kinase 1), TAB1 (TGF-β Activated Kinase 1 Binding Protein 1), TAB2 and TAB3 [24]. In a further step, TAK1 is activated by TRAF6 [55]. Once activated, TAK1 phosphorylates the IKK complex [56]. Next, the IKK complex phosphorylates the NFκB inhibitory protein IκBα, allowing the nuclear translocation of NFκB and the subsequent expression of proinflammatory genes [57]. Besides activating NFκB, TAK1 also phosphorylates MAPKs (p38, JNK and ERK), amplifying the inflammatory response. 

After early TLR4 activation, the receptor is redirected towards the endosome and promotes a late phase of NFκB activation and IFN production [58]. Innate immune cells efficiently use endocytosis to degrade microorganisms in the lysosomal compartment. Additionally, pathogens routinely use the endocytic pathway to gain access to the cytosol. Therefore, endosomes are a perfect place to find immune receptors, such as TLR4. CD14 plays a key role in LPS-mediated TLR4 endocytosis [59]. In this case, the TIRAP-MyD88 complex is released from the invaginated cell membrane, allowing TRIF (TIR-domain-containing adaptor protein inducing IFNβ) and TRAM (TRIF-related adaptor molecule) to engage the TIR domain of TLR4. Then, TRAF3 recruited by TRIF interacts with IKK-related kinases for IRF3 phosphorylation and further dimerization [60]. AIRF3 dimers translocate into the nucleus to induce the expression of type I IFN genes [61]. TRIF also participates in recruitment of TRAF6 and the kinase RIP-1, which activates the TAK1 complex. This leads to late activation of NFκB and MAPKs, with the subsequent inflammatory cytokines gene expression [48]. Remarkably, NFκB activation shows a delayed peak in Myd88-deficient cells compared with wild-type cells, supporting a late, Myd88-independent pathway of NFκB activation [62].

## 6. TLR4 Mediated Effects

TLR4 is a key molecule involved in the pathogenesis of inflammatory diseases [63,64]. Indeed, reports on PAMPs recognition mediated by TLR4 strongly support its role against different pathogens, mediating both secretion of proinflammatory cytokines and chemotactic factors that mediate the local recruitment of immune cells, including neutrophils and macrophages [46,65,66,67]. Additionally, TLR4 recognition of DAMPs in damaged tissues further contributes to local inflammation and fibrosis [68]. The damaged epithelial and endothelial cells produce cytokines and chemokines that contribute to attract immune cells to the wounded tissue [69].

Upon infection, bone marrow (BM)-derived myeloid cells are produced and then recruited to damaged tissues where they differentiate to dendritic cells and macrophages, contributing to the initial local inflammatory response [70,71]. Additionally, BM-derived monocytes with a patrolling profile are abundant during the late stages of local inflammatory processes and participate in its resolution and tissue repair, but also may produce fibrosis [64,65,71]. Alterations in vascular permeability mediated by TLR4 interactions allow infiltration of circulating cells in the injured tissues. Endothelial TLR4 is involved in neutrophil recruitment during LPS-induced systemic sepsis [72]. Extracellular histones, released from necrotic tubular epithelial cells during AKI, induce the secretion of the proinflammatory cytokines by BM–derived dendritic cells through TLR2 and TLR4 recognition [73]. Glucose induces TLR4 expression in podocytes and tubular cells and increases inflammation, renal injury and fibrosis in diabetes nephropathy, effects that were not observed in TLR4 deficient mice [74].

TLR activation alters the renal redox homeostasis [75,76,77]. In the kidney, accumulation of ROS (reactive oxygen species) and RNS (reactive nitrogen species) induces renal dysfunction [78] as well as increased TLR4 and Hsp70 expression by tubular cells [79]. TLR4/NLRP3 and TLR4-NK-κB are the principal pathways able to modulate the mitochondria-related oxidative damage. Different studies have demonstrated that TLR4 knockout mice are protected from renal injury since they have lower levels of chemokines and less granulocytic recruitment [38]. In the case of MyD88 and TRIF deficient mice the results are controversial [80].

In the first hours after AKI, TLR4 plays a fundamental role in the induction of adhesion molecules by vascular endothelium [36], facilitating infiltration of leukocytes to renal parenchyma [81]. TLR4 activation is also involved in endothelial dysfunction, causing abnormal vascular tone, hyperpermeability and proteinuria [82,83]. All these cellular dysfunctions may lead to important alterations in the microcirculation after TLR4 activation [84]. However, although inhibition of TLR4 improved renal function in a pre-clinical study, this protective effect was independent of restoration of macro-circulation or micro-circulation [85].

Renal damage induces an inflammatory response and apoptotic mechanism [86,87]. In the initial phase of tissue damage, renal tubular epithelial cells express markers of early apoptosis (caspase-8 and Bax, among others) [88]. In sepsis conditions, TLR2−/−, TLR4−/− and MyD88−/− deficient mice, present reduced renal apoptotic rates compared to wild type mice, favoring clearance of damaged cells [80]. Furthermore, in MyD88−/− mice, the anti-apoptotic bcl-2 protein is elevated compared to wild type mice, which results in protection from death [82]. In vitro analyses confirmed the notion that TLR4/MyD88/NF-κB axis regulates inflammatory response and apoptosis after renal injury [89]. In the context of renal damage, TLR4 can be activated by alarmins (endogenous ligands for TLR4; i.e., HGMB1 and Hsp70) as a consequence of cellular stress, amplifying kidney injury [90]. Both alarmins are elevated in septic mice (wild type, TLR2−/−, TLR4−/−), but not in the case of MyD88−/− mice [82]. In human biopsies, enhanced HGMB-1 is detected after renal graft ischemic transplantation [91]. Increased expression of KIM-1 (kidney injury molecule-1) and mTOR (mammalian target of rapamycin) was found in sepsis-associated AKI. These molecules are important downstream mediators of TLR4 activation in this pathological scenario [82,92]. 

## 7. Regulation of the TLR4 Pathway

TLR4 is essential for triggering an inflammatory response after PAMPs and DAMPs recognition. However, excessive, uncontrolled and/or sustained activation of these TLRs may lead to a chronic inflammatory state that promotes the development of several immune-mediated diseases. Therefore, strict negative regulation of TLR4 signaling is required to protect the host from an exacerbated inflammatory response. Modulation of TLR4 activation and their downstream-related signaling pathways include several mechanisms, such as soluble decoy receptors, transmembrane regulators, cellular trafficking, destabilization of adaptor proteins, ubiquitination, dephosphorylation, transcriptional regulation and feedback inhibition (Table 2). 

Several studies have reported the existence of soluble forms of receptors that act as decoy for ligands and coreceptors of transmembrane receptors themselves, thus preventing ligand-receptor interaction and their subsequent activation. This is the case of the soluble form of TLR4 (sTLR4). It has been demonstrated that recombinant sTLR4 significantly reduced NFκB activation and TNFα production in vitro [93] through its association with TLR4 ligands and coreceptors like MD2 and CD14 [84]. The assembly with sTLR4 prevented the interaction of ligands and coreceptors with transmembrane TLR4, therefore efficiently attenuating TLR4 activation. 

Negative modulation of TLR4 also involves transmembrane proteins with an intracellular TIR domain, such as ST2L and SIGIRR. Through this TIR domain, ST2L and SIGIRR can bind to MyD88 and IRAK and, as a result, hinder their recruitment after TLR4 activation [95,96]. Increased levels of ST2L has been reported in infection- and obstructive-associated renal injury [113]. SIGIRR deficiency increased renal proinflammatory cytokine levels and aggravated post-ischemic AKI [114]. However, lack of SIGIRR did not prevent postobstructive renal fibrosis [43]. There are other transmembrane proteins without TIR domain that also have an inhibitory effect on TLR4. For example, RP105 associates directly with the TLR4 extracellular domain, avoiding ligand binding to this receptor, thereby inhibiting TLR4 signaling [97]. 

TLR4 subcellular location also represents a target for negative regulation of the receptor. From the cell surface, TLR4 activation induces a MyD88-dependent pathway, while its endocytosis triggers a MyD88-independent pathway. Different proteins modulate TLR4 internalization and trafficking and promote signaling through one of these pathways [115]. After stimulation, Rab7 protein leads TLR4 to the lysosomal compartment for degradation, preventing its activation and favouring its turnover [98]. A recent study has reported elevated levels of Rab7 after ischemia-reperfusion kidney injury. However, the authors have related this elevation to autophagy activation after renal injury, independently of the acute inflammatory response triggered by TLRs [116].

Intracellular proteins recruited after TLR4 activation (Myd88, TRAF6, IRAK and TIRAP) are involved in modulation of TLR4 signaling. Several adaptors variants act as negative regulators, thus preventing downstream signaling. One of these adaptor variants is MyD88s, an alternative form of MyD88 without the interdomain. MyD88s heterodimerizes with MyD88, inhibiting MyD88-IRAK4 interaction and preventing subsequent IRAK4-mediated IRAK1 phosphorylation, decreasing downstream signaling [99]. IRAK1 is also modulated by TOLLIP, which reduces its autophosphorylation and favours its ubiquitination, inhibiting post-IRAK1 signaling [100]. In a sepsis model of renal injury, TOLLIP inhibited LPS-induced TLR4 signaling by suppressing IRAK1 activation [117]. TOLLIP gene expression was reduced in experimental ischemia-reperfusion AKI [118]. Another key modulator in TLR4 signaling is SOCS1 (Suppressor of Cytokine Signaling 1). SOCS1 is induced upon receptor activation and modulates TLR4 through two mechanisms. First, SOCS1 interacts directly with TIRAP and initiates its degradation through ubiquitination. Second, SOCS1 directly associates with p65 subunit of NFκB and induces its proteasomal degradation, thereby suppressing the activation of NFκB [101]. Induction of Jak2/STAT3/SOCS1 pathway had a protective effect in acute kidney graft rejection [119]. In a murine model of cisplatin-induced AKI, SOCS1 expression was decreased, whereas induction of Jak2/STAT1 pathway with an AMPK activator restored SOCS1 levels and promoted renal protection [120]. Additionally, SOCS1-targeted therapy limited progression of diabetic nephropathy [121,122,123].

Ubiquitination and dephosphorylation of TLR4 or the proteins involved in TLR4 signaling is essential for the modulation of this signaling pathway. For example, the deubiquitinating enzyme A20 inhibits TLR4 mediated signaling by deubiquitination of TRAF6 and therefore blocks effectively MyD88 dependent and independent pathways [102]. Induction of A20 in renal proximal tubular epithelial cells showed an anti-inflammatory and anti-apoptotic effect [124]. In ischemia-reperfusion renal injury, A20 has also a protective role by suppressing pro-inflammatory pathways [125,126]. Moreover, TRIAD3A promotes ubiquitination and subsequent degradation of TLR4 receptor itself [103]. Overexpression of TRIAD3A was reported in diabetic nephropathy [127]. Additionally, TLR4 signaling can be limited by the tyrosine phosphatases SHP1 and SHP2. These enzymes catalyze the dephosphorylation and consequent inhibition of IRAK1 and TBK1, respectively. In a recent study, SHP1 inhibited renal ischemia-reperfusion injury by dephosphorylating ASK1 and suppressing apoptosis. Consistently, SHP1 knockdown mice showed significantly increased renal injury and aggravated the apoptosis of tubular epithelium cells [128]. Controversially, genetic deficiency or inhibition of SHP2 seems to have a beneficial effect in kidney damage. PHPS1, an inhibitor of SHP2, attenuated renal injury in a murine model of hemorrhage followed by sepsis [129]. SHP2 knockout in myeloid cells protected the kidneys from inflammatory damage and prevented renal fibrosis after unilateral ureter obstruction [130]. SHP2 deficiency also had a protective effect in renal ischemia-reperfusion [131]. SHP2 is assumed to have an additional role as an antagonist of TBK1 beyond its phosphatase activity [104,105]. Ligand binding leads to phosphorylation of tyrosine residues within the TIR domain of TLR4. This phosphorylation is required for TLR4-induced activation of NFκB. Although the mechanisms are not completely understood, tyrosine kinases like Src and Btk modulate TLR4 phosphorylation state by binding to the receptor after its activation. Inhibition of these kinases reduce TLR4 phosphorylation and signaling [115].

TLR4 signaling can also be modulated by reducing the expression of this receptor. The expression of TLR4 can be downregulated by TGF-β and the anti-inflammatory cytokine IL-10. Specifically, TGF-β inhibits TLR4 gene expression and promotes MyD88 degradation, thus decreasing downstream signaling [106,107]. On the other hand, IL-10, throughout miR-146b, reduces the expression of TLR4, MyD88, IRAK1 and TRAF6 [108]. Several studies have identified other miRNAs involved in the modulation of these molecules. For example, miR-155-5p reduces the expression of MyD88 and causes the abrogation of NFκB activation. miR-155-5p is highly expressed in kidneys from renal patients and experimental obstructive renal models, promoting renal fibrosis [132]. However, the relationship between miR-155-5p and acute renal inflammation has not been reported and further studies are required. TLR4 activation increases the expression of miR-210-5p, which also regulates the activation of NFκB pathway throughout inhibition of NFκB1 mRNA subunit [109]. 

Feedback inhibition of TLR4 contributes significantly to the overall control of the downstream signaling. Different studies support the existence of various proteins that are induced after TLR4 activation and that negatively modulate this signaling pathway. TRIM30α, a member of TRIM protein superfamily, is induced after TLR4 ligand recognition and activation. This protein enhances the degradation of TAB2 and TAB3 and effectively inhibits NFκB activation [110]. TLR4 also increases the expression of other negative modulators, including IκBNS and Bcl3. IκBNS binds to IL-6 promoter and reduces its induction [111]. Bcl3 attenuates the inflammatory response by associating with the DNA binding site of NFκB p50 subunit, limiting its action [112]. Bcl3 expression was reported upregulated in experimental AKI [133]. In vitro Bcl3 overexpression decreased NFκB transcriptional activity, inflammation and cell death, whereas its downregulation resulted in chemokine upregulation, sensitization to cell death and increased NFκB transcriptional activity [133]. The aforementioned molecules and mechanisms represent promising targets for the design of new therapeutic strategies against exacerbated TLR4-inflammatory response. 

## 8. TLRs and AKI

**Figure 2:**
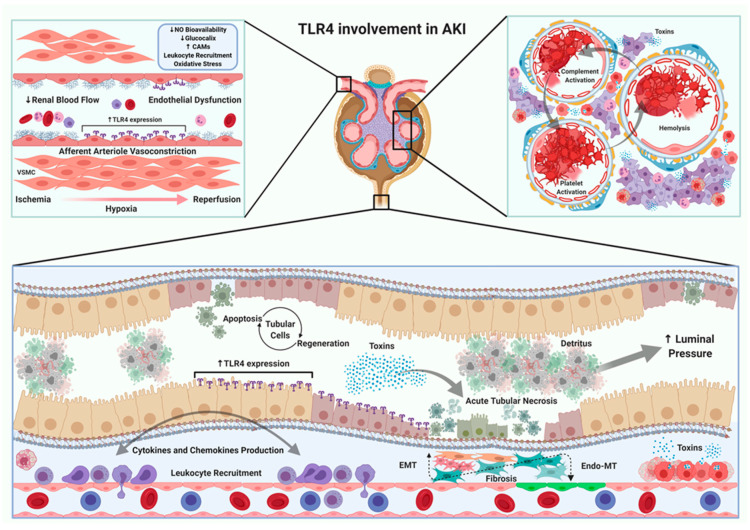
TLR4 in the pathophysiology of AKI. Activation of TLR4 signaling pathway has been observed in different types of AKI. On the one hand, models that reduce renal blood flow generate an increase of TLR4 expression in endothelial, tubular and leukocyte cells. Endothelial dysfunction is a common mechanism in all types of AKI. This dysfunction leads to an increase in the expression of cell-adhesion molecules, activation of the innate immune response (neutrophils and macrophages) and the concomitant maintenance of oxidative stress. On the other hand, the presence and/or release of endo/exotoxins in some AKI, allows the development of endothelial dysfunction as well as activation of platelets and complement cascade, which obstruct glomerular blood flow, decreasing glomerular filtration rate. Other pathological changes detected in different types of AKI are largely observed in the tubulointerstitial space, mainly related to accumulation of toxins. The maintenance of a partial and/or total obstruction in the urinary tract, either by an exogenous (e.g., prostatic hyperplasia) or endogenous mechanism (e.g., detritus overload), produces an increase in luminal pressure, dilation of the collecting ducts, loss of functional units and development of tubulointerstial fibrosis. The pre-renal, intrarenal and post-renal manifestations are closely linked and the affection of one compartment has a direct impact on the others, enhancing the severity of AKI. Created with BioRender.com.

There are multiple causes of AKI. For that reason, in the next section we will fully describe the role of TRLs, mainly TLR4, in each specific AKI subtype (Figure 2).

### 8.1. Ischemia-Reperfusion

Ischemia reperfusion (I/R) injury is caused by a period of vascular occlusion followed by blood supply to tissue. Blockade of blood flow induces hypoxia and accumulation of metabolic products in the tissue [134]. Ischemic injury may be classified as warm, in the case of vascular anastomosis, stroke or myocardial infarction, or cold ischemic injury, as reported in tissue transplantation. The kidney is extremely sensitive to warm ischemia, but relatively resistant to prolonged periods of cold ischemia [135]. The key problem in the ischemic process is the subsequent period of reperfusion, which causes severe renal injury by activating the innate inflammatory response [136,137,138].

Several studies have described a deleterious role of TLRs in AKI induced by I/R. The TLR3/TRIF/IRF-3 signaling pathway is activated in experimental I/R-associated AKI [139]. In the same article, TLR3 knockout mice showed decreased renal injury, preserved renal perfusion, and reduced renal inflammation as compared with wild type mice, suggesting that TLR3 is involved in renal IR injury [139]. Increased TLR2 and TLR4 gene expression was reported in proximal and distal tubules after I/R damage [37]. 

Some ligands of TLR2 and TLR4 such as hyaluronan, HMGB1 and brevican were also elevated in this pathological setting [38]. Interestingly, the treatment with an antibody against HMGB1 reduced recruitment of neutrophils and macrophages and diminished inflammation and apoptosis in TLR4+/+ mice, but not in TLR4−/− mice, indicating the key role of HMGB1/TLR4 axis in I/R injury [140]. A recent study identified miR-27a as a negative regulator of TLR4 and demonstrated that overexpression of this miRNA reduced TLR4 expression and consequently diminished I/R-mediated renal inflammation and cell death [141]. On the other hand, the in vivo gene blockade of TLR2, TLR4 or MyD88 demonstrated beneficial effects against renal I/R injury, including reduction of inflammatory infiltrate, decreased epithelial cell death, reduced expression of cell adhesion molecules, and amelioration of renal function [36,38,80,142]. In other study, TLR2, MyD88, TRIF, and MyD88/TRIF knockout mice were protected against I/R damage [143]. The in vivo experiments developed by Rusai et al. described non-synergistic beneficial effects in TLR2 and TLR4 double knockout mice submitted to I/R as compared to single gene deletion [144]. Induction of I/R injury in TLR4 knockout mice also protected from microvascular rarefaction but not from the development of fibrosis [145]. A more severe injury after I/R was observed in mice with targeted deletion of complement factor B and TLR2 as compared to mice with single factor B deficiency [146]. Induction of hypoxia in cultured proximal tubular epithelial cells demonstrated the stimulation of the endoplasmic-resident gp96, a molecule that physically interacts with TLR4. This tandem is necessary for the activation of ASK1/JNK signaling pathway. In addition to these data, NOX4 collaborated with TLR4-mediated apoptosis in renal I/R [147]. Kidney transplantation is a pathological situation closely related to I/R damage. In this setting, TLR2 or MyD88 gene deficiency improved renal function and reduced chronic allograft damage by reducing cytokine and chemokine expression (IL-6 and CCL2), leukocyte recruitment and fibrosis (collagen I and III levels) in renal grafts [148]. A study of I/R AKI with mice deficient in TLR9 in tubular cells reported an improvement in renal function, an effect not observed in total TLR9 knockout mice, suggesting the complex role of this protein in this pathology [149]. Treatment with a TLR9 antagonist, reduced renal damage in mice [150]. Furthermore, activation of TLR5 with its agonist CBLB502 reduced acute renal ischemic failure lesions [151].

Some compounds targeting TLRs expression have demonstrated beneficial effects against I/R injury. The anti-inflammatory Maresin 1 reduced renal damage through the modulation of TLR4 and ERK, JNK, and P38 signaling pathways in I/R mice [152]. Hesperidin also ameliorated renal function and reduced inflammatory mediators, oxidative stress, as well as TLR4 and NFκB activation [153]. Eritoran is a molecule that inhibits TLR4 dimerization [154] and attenuated the course of I/R injury by decreasing serum creatinine, tubular damage markers (KIM-1, N-GAL), monocyte infiltration and expression of IL-6, TNF-α and IL-1β [155]. Besides its effects targeting Nrf2, sulforaphane specifically suppresses oligomerization of TLR4 and decrease inflammatory response [156]. In an acute model of I/R, sulforaphane reduced renal injury, inflammation, oxidative stress and cell death [157]. All together, these data demonstrate the essential role of TLRs in I/R injury as well as kidney transplantation.

### 8.2. Toxin Induced AKI

#### 8.2.1. Endogenous Toxicity (Pigment Nephropathy)

Excessive accumulation of endogenous toxins in the kidney may lead to AKI. This is the case of rhabdomyolysis, a pathological condition characterized by massive muscle injury and the consequent release of intracellular content into the bloodstream, mainly myoglobin and heme-derivates. These molecules build up in the kidney, resulting nephrotoxic and promoting AKI [158]. Heme directly activates TLR4, so TLR4 may play an important role in rhabdomyolysis-induced AKI [159]. 

TLR4 has been proposed as a biomarker of rhabdomyolysis-AKI. Thus, TLR4 was found in the urine as a consequence of protein cleavage and massive proximal tubular cell death [160]. By contrast, low TLR4 levels were found in kidneys, probably due to tubular necrosis [160]. Pharmacological inhibition of TLR4 with TAK-242 suppressed myoglobin-induced inflammatory response in cultured macrophages and tubular epithelial cells [161,162]. TAK-242 inhibits TLR4 interaction with its adaptor molecules [163]. This small-molecule inhibitor decreased inflammatory cytokines production, macrophage infiltration and renal damage in a rhabdomyolysis model induced by intramuscular injection of glycerol [161]. In other study, rhabdomyolysis was induced by exerting 3 kg of pressure for 8 h in rats, and treatment with TAK-242 reduced renal injury and decreased systemic inflammatory cytokines (IL-6 or TNF-α) [164]. In line with this data, other studies also reported that direct inhibition of the TLR-4/JNK/p38/NFκB pathway with curcumin and loganetin [165,166], reduced kidney injury and inflammation, as well as improved renal function in pre-clinical models of rhabdomyolysis [45,165]. However, there are contradictory results in the literature, since TLR4 antagonism with TAK-242 did not protect from rhabdomyolysis-mediated renal damage in other study [162]. Therefore, new studies are necessary to address the contribution of TLR4 in rhabdomyolysis. 

#### 8.2.2. Exogenous Toxicity 

The development of AKI can be a side effect of some pharmacological therapies. Cisplatin, a drug used for the treatment of certain tumors and may induce AKI [167]. TLR4 has been associated with cisplatin-induced AKI. Indeed, TLR4-knockout mice were protected from kidney damage and showed lower levels of inflammatory cytokines and chemokines than wild type mice after cisplatin administration [40,168,169]. In this line, the TLR4 inhibitor sulforaphane attenuated cisplatin-induced renal dysfunction, histological damage and oxidative stress [170].

Certain antibiotics, such as aminoglycosides, also lead to AKI. There are fewer data about the involvement of TLR4 in this type of AKI. However, two recent studies described that pretreatment with pirfenidone or umbelliferone caused a reduction in renal damage through the inhibition of the TLR4/NFκB/NLRP-3 pathway in an experimental model of gentamicin-induced AKI [171,172].

Another nephrotoxic drug is acetaminophen [173]. TLR4 has been suggested as a possible target against the organ failure caused by acetaminophen. Thus, TLR4 inhibition with TAK-242 decreased kidney injury and improved renal function, but this study does not clarify whether this protective effect is directly on the kidney or indirectly through the hepato-renal crosstalk [174]. The involvement of TLR4 in the hepato-renal syndrome has also been observed in experimental models, where increased renal TLR4 expression has been described, mainly in tubular cells, and related to kidney injury [175]. This fact has also been observed in cirrhotic patients that developed AKI [176].

Radiocontrast medium used in medical tests can lead to AKI [177]. Increased renal TLR4 expression was observed after injection of contrast medium [178]. In this contrast medium-induced AKI model, TAK-242 protected against tubular apoptosis and ROS production by modulating NLRP3 inflammasome. These results were reproduced in cultured rat kidney cells (NRK-52e), where TAK-242 decreased iopromide-mediated cell death, oxidative stress and inflammation [178]. 

### 8.3. Sepsis-Induced AKI

Sepsis is a life-threatening pathological condition associated to a systemic infection and that may lead to AKI (Sepsis-induced AKI (SI-AKI)) [179]. Despite a considerable number of studies in recent years, the pathophysiology of SI-AKI is not fully understood, and current treatment is limited to replacement of renal function by dialysis. In the past, SI-AKI was related to hypoperfusion and the subsequent renal ischemia. However, recent studies suggest that SI-AKI can also occur within normal blood pressure range and maintained renal perfusion [84]. According to recent experimental and clinical studies, pathophysiology of septic AKI is explained through three main mechanisms: alterations in renal microcirculation related to endothelial dysfunction, inflammation, and adaptive bioenergetic and metabolic downregulation in renal tubules [84]. This recent paradigm shift highlights the role of the inflammatory response in SI-AKI and points strongly towards TLR4 as a potential mediator in the development of SI-AKI. LPS is the main ligand for TLR4 [180]. The interaction between LPS and both systemic and renal TLR4 has been reported in SI-AKI [181]. After polymicrobial sepsis, expression of TLR4 increases in renal tubules, glomeruli and vasculature [39] and circulating endotoxins have been detected in these locations [182]. Recent animal studies have demonstrated that targeting TLR4 with specific antibodies reduce endotoxemia-associated mortality [183,184]. To further reinforce the potential role of TLR4 in SI-AKI, C3H/HeJ mice characterized by a dysfunctional TLR4 showed an attenuated renal injury when subjected to LPS [87]. In humans, TLR4 polymorphisms have been related with a reduced LPS-mediated inflammatory response [185]. 

Based on the aforementioned data, different studies have been performed in order to investigate whether TLR4 inhibition may have a renoprotective effect in SI-AKI. In a sheep model of SI-AKI induced by intravenous LPS infusion, the selective TLR4 inhibitor TAK-242 reduced plasma creatinine and BUN concentration [186]. TAK-242 also reduced sepsis-mediated glomerular neutrophil infiltration and endothelial swelling, improved creatinine clearance, glomerular filtration rate and urine output [85]. In a cecal ligation and puncture model of SI-AKI, TLR4 knockout mice showed preserved renal morphology and function, decreased vascular permeability and lower neutrophil infiltration in the kidneys, as well as reduced IL-1β, TNF-α, IL-6 and IL-17 levels in the kidney and in the peritoneal cavity, with a marked decrease in NFκB activation [82]. TAK-242 attenuated LPS-mediated renal dysfunction and pathological damage by inhibiting TLR-4/MyD88/NFκB signaling pathway [187]. TAK-242 also ameliorated LPS-mediated pro-inflammatory cytokine expression and reduced NFκB activation in cultured renal epithelial cells [188,189]. Moreover, urinary inflammatory markers NGAL and IL-18 were also reduced by TAK-242 administration in I/R injury [190]. Resveratrol, a natural phytoalexin, also reduced TLR4 expression and NFκB activation in macrophages and mice with LPS-induced AKI [191]. In SI-AKI, hydrogen sulfide also reduced oxidative stress and pro-inflammatory cytokines expression via the TLR4/NLRP3 signaling pathway [78,192]. Moreover, TLR4-mediated expression of cell adhesion molecules (ICAM-1 and E-selectin) may contribute to renal leucocyte infiltration and renal injury in SI-AKI [193,194]. 

Different studies have tried to determine the relative importance of renal and systemic TLR4 in the recruitment of immune cells and renal injury in SI-AKI. In an animal model of SI-AKI by LPS injection, wild type mice received transplanted kidneys from the C3H/HeJ LPS-hyporesponsive mouse strain and developed renal inflammation and AKI after LPS exposure, while C3H/HeJ with kidneys from wild type mice did not show these alterations [87]. Therefore, this study emphasizes the essential role of systemic TLR4 for the development of SI-AKI. In contrast, a more recent study supports the importance of renal TLR4 in SI-AKI. The endotoxin increased tubular injury in mice without TLR4 in hematopoietic cells but were TLR4 +/+ in renal cells. Conversely, mice with TLR4+/+ immune cells and TLR4−/− in renal cells had no tubular injury [195]. In addition to these studies, results derived from a mouse model of pyelonephritis also using chimeric mice suggest that both systemic and renal TLR4 are necessary for abscess formation and leukocyturia in this model [196]. Even though it remains unclear whether the renal or systemic TLR4 has a more important role, all these studies share the common protective effect of the lack of TLR4 in the development of SI-AKI.

Beyond inflammation, SI-AKI is characterized by two additional pathological mechanisms: dysregulated renal microcirculation and metabolic adaptation of renal cells to injury. Although the connection with TLR4 might not be direct, as in the case of the inflammatory response, recent evidence suggests the potential beneficial effect of TLR4 inhibition against these harmful processes. Endothelial dysfunction in sepsis is characterized by dysregulated renal blood flow and reduced GFR [197]. Pharmacological inhibition or genetic deletion of TLR4 in pre-clinical sepsis models was associated with reduced glomerular endothelial swelling and vascular permeability, respectively [82,85]. In a recent work LPS-mediated reduction in GFR was abolished in TLR4 knockout mice, suggesting a TLR4-dependent mechanism [198]. Tubular cells exposed to inflammatory stimuli prioritize cell survival over organ function [199]. Downregulation of renal sodium and chloride transporters after LPS exposure has been previously described [197]. However, recent reports have demonstrated that TLR4 signaling pathway is also involved in LPS-mediated inhibition of HCO_3_(−) absorption in the kidney [92]. According to all these data, it is not possible to deny the potential therapeutic effect of TLR4 inhibition against the complex pathological consequences of SI-AKI (Table 3). However, it is important to keep in mind that TLR4 activation and its consequent pro-inflammatory response is necessary for bacterial elimination during sepsis. 

Other members of the TLRs family have been involved in SI-AKI. For example, TLR2 was overexpressed in glomerular endothelial cells and podocytes in a murine model of SI-AKI [200]. TLR2 knockout mice subjected to cecal ligation and puncture (CLP) had preserved renal morphology, less renal hypoxia, fewer areas of apoptosis, reduced expression of proinflammatory cytokines and decreased vascular permeability compared with wild type animals [82]. Another study has reported that histones released from dying renal cells in AKI directly interact with TLR2 and TLR4. Extracellular histones induced leukocyte recruitment, microvascular leakage and renal inflammation in a TLR2/TLR4 dependent manner [73]. Flagellin, the monomeric subunit of bacterial flagella, triggers innate immune response through TLR5. A recent work compared the systemic effect of LPS and flagellin administration in mice. Flagellin induced significant oxidative stress and liver but not renal injury, whereas LPS caused less severe oxidative stress and triggered renal but no hepatic damage [201]. Based on this work, the contribution of TLR5 to SI-AKI seems modest. Beyond bacterial infections, AKI is also associated with severe influenza infections in patients. In this regard, TLR7 activates B lymphocytes and contributes to the development of glomerulonephritis in response to viral agonists [197,202]. TLR9 recognizes, among other ligands, viral and bacterial DNA motifs. Induction of TLR9 expression was reported in mouse kidney tissue after CLP [203]. Silencing of renal TLR9 with siRNA reduced cell apoptosis, attenuated the severity of AKI and increased the survival of mice after SI-AKI induced by CLP [203]. TLR9-knockout mice have a reduced cytokine production, splenic apoptosis, and kidney injury in SI-AKI [204]. Furthermore, TLR9 knockout mice have lower levels of IL-17A and IL-1β after cecal ligation and puncture producing SI-AKI [205]. Administration of chloroquine, an inhibitor of endocytic TLRs (TLR3, TLR7, TLR8, TLR9), reduced sepsis-induced mortality and renal injury severity in SI-AKI. In the same study, TLR9 genetic and pharmacological inhibition mirrored the protective effect of chloroquine administration [206].

### 8.4. Thrombotic Microangiopathy-Induced AKI

One of the most common causes of AKI in children is the infection with Shiga toxin (Stx)-producing *Escherichia coli* [218]. Stx causes hemolytic uremic syndrome (HUS), which is characterized by thrombotic microangiopathy and severe renal damage [219]. TLR4 has been identified as the specific receptor of Stx in human neutrophils [220], and its presence increases the expression of TLR4 in renal cells [221]. Similarly, peripheral blood monocytes from HUS patients with Stx showed higher levels of cell surface TLR4, as well as enhanced LPS-mediated inflammatory response than control individuals [222]. However, the soluble TLR4 extracellular domain also inhibits the interaction between Stx and human neutrophils [223]. Therefore, the binding of TLR4 to Stx may have a protective role by sequestering the toxin, or a harmful role by being a direct receptor of Stx, increasing its toxicity. In a recent study, Stx increased creatinine levels in wild type and MyD88 knockout mice, but not in TLR4 knockout mice, suggesting a crucial role of TLR4 in Stx-induced kidney disease [224]. 

## 9. AKI to CKD Transition

**Figure 3:**
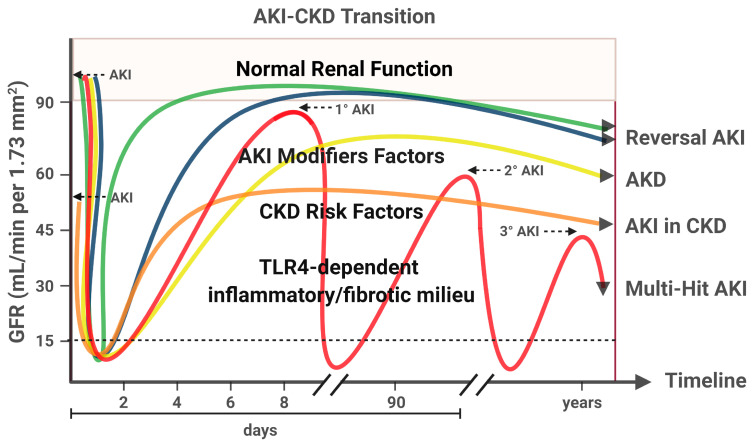
AKI-CKD Transition. There are multiple renal outcomes after AKI, from full recovery of renal function to chronic damage and permanent renal impairment. The lines on the graph represent the variation in glomerular filtration rate (GFR) during an AKI event, according the current definitions by KDIGO-AKI workgroup. The green and blue lines show a favorable scenario after an AKI event, with full recovery of renal damage and no evidence of CKD. An important difference between these lines is a better outcome in the case of an early improvement (<2 days, green line). The yellow line shows the persistence of acute/subacute kidney damage between the 7th and 90th day after the initial AKI event, currently called Acute Kidney Disease (AKD). The orange line shows an AKI event in the context of CKD. Patients suffering from AKI, in the presence of CKD, have a higher risk of progressing to chronic renal damage. The red line shows recurrent AKI events in absence of previous CKD. These multiple injury hits significantly reduce GFR over time. AKI episodes may be more intense or recurrent in genetically susceptible individuals and in the presence of other risk factors, including an established CKD. Growing evidence supports that TLR4-dependent pro-inflammatory/pro-fibrotic effects may play a key role in AKI-CKD transition. Created with BioRender.com.

AKI can actively contribute to the development of CKD, with the subsequent increase in cardiovascular risk and death [225,226]. AKI to CKD transition is associated with incomplete recovery of renal function over time (Figure 3). This adverse outcome is more frequently observed in CKD patients. Other prognostic factors involved in AKI to CKD transition include age, gender, severity, recurrence and duration of AKI, as well as classical CKD risk factors (diabetes, hypertension, obesity and proteinuria) [15]. Although the AKI to CKD transition is a continuum, the KDIGO-AKI workgroup has recently defined a new concept, acute kidney disease (AKD), as the persistence of acute or subacute kidney damage greater than 7 days, but less than 90 days, after an initial AKI event, characterizing an intermediate pathological stage between AKI and CKD [15].

Induction and maintenance of AKI-CKD transition depends on inflammatory and fibrotic mediators expressed during initial stages of AKI [227,228,229]. AKI insults promote tissue damage and cell death, resulting in an early acute inflammatory response, and subsequent reparative and regenerative reactions in late phases of AKI to restore renal parenchyma [230,231]. However, persistent inflammatory response may conduct to the development of fibrosis after AKI [227]. This fibrotic process is mediated by phenotypic and functional changes of epithelial and endothelial cells toward a mesenchymal state, the so-called epithelial to mesenchymal transition (EMT) and endothelial to mesenchymal transition (Endo-MT). AKI-associated fibrosis is also related to the appearance of myofibroblasts or fibroblast-like phenotype in the kidney [232,233]. Therapeutic targets that limit the progression of EMT and Endo-MT may play a relevant role in the progression of AKI-associated kidney damage [234,235].

There are some studies that link TLRs with fibrosis and AKI to CKD transition [236]. TLR4 has been associated with fibroblast differentiation during AKI [237,238]. Moreover, TLR4-deficient mice were protected against renal fibrosis due to a low expression of α-smooth muscle actin at the tubulointerstitial level [238]. Systemic administration of folic acid in mice has been used for studying the pathogenesis of AKI eliciting renal fibrosis and favoring AKI-CKD transition [239]. TLR4 knockout mice protected against the appearance of fibrosis after folic acid injection [237]. Similar results were observed after pharmacological inhibition of the TLR4 pathway [240,241].

## 10. Clinical Trials Targeting TLR4 in AKI

Many drugs targeting TLR4 are currently being evaluated in phase II and III clinical trials in a variety of inflammatory pathologies, such as rheumatoid arthritis, nonalcoholic steatohepatitis, insulin sensitivity as well as Myelodysplastic syndrome [242]. Some of these TLR4 inhibitors have strong anti-inflammatory effects and prevent cytokine production in these diseases, such as Eritoran, NI-0101, CX-01 and JKB-121 [242]. This is why TLR4 signaling pathway has received great interest from researchers in the nephrology field. 

The effects of specific TLR4 inhibitors have been analyzed in many preclinical AKI models [243]. As previously reported, several natural (loganetin, resveratrol and curcumin) and synthetic compounds (TAK-242, Eritoran and hydrogen sulfide) showed beneficial effects in experimental models of AKI (Table 3). Surprisingly, despite these positive results, at present, there is no data of clinical trials targeting TLR4 in patients suffering from AKI. There is only a clinical trial that analyzed the effect of the anti-inflammatory compound pirfenidone on renal function in septic AKI (NCT02530359). The main outcomes include mortality, renal function, inflammation and TLR4 circulating levels. However, results are currently pending. 

## 11. Perspectives and Conclusions

TLR4 plays an important role in innate immunity via PAMPs and DAMPs recognition. However, exacerbated activation of the TLR4 signaling pathway promotes harmful effects in many tissues, including kidney. There is a close relationship between TLR4 and AKI development. A number of preclinical studies have demonstrated that TLR4 gene deficiency or inhibition ameliorated renal function, decreased histological damage and reduced inflammation, oxidative stress and cell death in different types of AKI. Moreover, TLR4 is also related to AKI to CKD transition by promoting fibrosis. However, to our knowledge, no clinical trials have been designed to specifically analyze whether TLR4 targeting is beneficial for patients with AKI. Further studies in humans are necessary to validate the potential favorable effect of TLR4 inhibition against AKI.

## Figures and Tables

**Table 1:** AKI biomarkers.

Biomarker	Function	Origin	Samples
Cystatin C	Extracellular cysteine protease inhibitor	All nuclear cells	Serum
NGAL	Limits bacterial growth by sequestering iron-containing siderophores	Neutrophils	Serum/Urine
IGFBP7	Main regulator of IGFs availability in cells	Renal and inflammatory cells	Urine
TIMP2	Peptidase involved in MMP inhibition and ECM degradation		
CCL14	Pro-inflammatory chemokine		
IL-18	Pro-inflammatory cytokine		
KIM-1	Phosphatidylserine receptor that recognizes apoptotic cells and oxidized lipoproteins	Tubular cells	
NAG	Hydrolytic lysosomal enzyme		
NHE3	Sodium–hydrogen exchanger in apical side of the epithelial cells		
α/π-GST	Phase II enzyme involved in cellular detoxification		
ALAP	Enzymes involved in tubular damage		
AP			
γGT			
L-FABP	Cytoplasmic proteins involved in binding, transport and metabolism of LCFAs		

Abbreviations: CCL14: Chemokine (C-C motif) ligand 14; ECM: Extracellular matrix; GST: Glutathione-S-transferase; IGF: Insulin growth factor; IGFBP7: Insulin-like growth factor-binding protein 7; IL-18: Interleukin-18; KIM-1: Kidney injury molecule-1; LCFA: Long-chain fatty acid; L-FABP: liver-type fatty acid binding protein; NAG: *N*-acetyl-β-D-glucosaminidase; NHE3: Sodium-hydrogen exchanger isoform 3; NGAL: Neutrophil gelatinase-associated lipocalin; MMP: Metalloproteinase; TIMP2: Tissue inhibitor of metalloproteinases-2.

**Table 2:** Mechanisms of negative regulation of TLR4 pathway.

Regulation	Regulator	Proposed Mechanism	References
**Soluble receptor**	sTLR4	Decoy for TLR4 ligands and coreceptors, preventing their association with the functional transmembrane receptor	[93,94]
**Transmembrane regulators**	ST2L	Bind to Myd88 and IRAK to reduce their recruitment to TLR4	[95,96]
	SIGIRR		
	RP105	Associates with TLR4 extracellular domain and prevents ligand binding	[97]
**Cellular trafficking**	Rab7	Promotes TLR4 endosomal internalization and degradation	[98]
**Adaptor proteins**	Myd88s	Heterodimerizes with Myd88 and inhibits its interaction with IRAK4	[99]
	TOLLIP	Reduces IRAK1 autophosphorylation and promotes its degradation	[100]
	SOCS1	Induces TIRAP and p65 degradation	[101]
**Ubiquitination**	A20	Promotes deubiquitination of TRAF6	[102]
	TRIAD3A	Induces TLR4 ubiquitination and subsequent degradation	[103]
**Phosphorylation**	SHP1	Dephosphorylates IRAK1	[104]
	SHP2	Dephosphorylates TBK1	[105]
**Transcriptional regulation**	TGFb	Inhibits TLR4 gene expression and promotes Myd88 degradation	[106,107]
	IL10	Reduces TLR4, Myd88, IRAK1 and TRAF6 expression	[108]
	miR-155-5p	Reduces Myd88 expression	[109]
	miR-210-5p	Inhibits NFkB1 mRNA subunit	[109]
**Feedback inhibition**	TRIM30α	Promotes TAB2 and TAB3 degradation	[110]
	IκBNS	Reduces IL6 gene expression	[111]
	Bcl3	Blocks p50 ubiquitination and prevents DNA binding of NFkB	[112]

**Table 3:** Specific compounds targeting TLR4 on experimental AKI.

Compound	AKI Model	Beneficial Effects	Reference
**TAK-242**	I/R	Reduces serum creatinine and urea concentration. Decreases IL-18 and malondialdehyde renal levels	[190]
	Rhabdomyolysis	Decreases functional and histological renal damage. Reduces renal TNF-α, IL-6 and IL-1β levels and macrophage infiltration	[161]
		Decreases renal damage, serum creatinine and BUN levels. Reduces renal IL-6, TNF-α and TLR4 expression and NFκB signaling	[164]
	Acetaminophen induced AKI	Decreases histological renal damage, serum creatinine concentration and partially restores glutathione levels	[174]
	Sepsis induced AKI	Improves creatinine clearance and urine output, reduces renal neutrophil accumulation and glomerular endothelial swelling	[85]
		Attenuates arterial pressure, plasma creatinine and BUN levels	[186]
		Reduces TLR4-Myd88-NFκB signaling pathway, decreases renal levels of TNF-α, IL-1β and IL-6 and inflammatory cell infiltration	[187]
**Eritoran**	I/R	Decreases serum creatinine, renal histological damage, monocyte infiltration and inflammatory markers (TNF-α, IL-1β, CCL2)	[155]
**Resveratrol**	I/R	Ameliorates histological renal damage, serum creatinine and BUN, reduces renal levels of IL-6, TNF-α, IL-10, IFNγ, caspase-3 activity and improves redox balance (MDA, SOD)	[137,138]
	Rhabdomyolysis	Attenuates creatinine levels, cortical macrophage infiltration and necrosis. Decreases renal NFκB activation as well as HO-1 and nitrotyrosine expression.	[207]
	Sepsis induced AKI	Improves renal function and tubular epithelial cell injury. Decreases serum content and renal mRNA expression of TNFα, IL-1β and IL-6. Inhibits IRE1 phosphorylation and NFκB activity in the kidney	[208]
		Improves renal function, reduces serum and renal inflammation, macrophage infiltration and TLR4 activation. Prevents endothelial cell permeability. Decreases expression of iNOS, Bcl2 and BclxL in macrophages	[191]
**Curcumin**	I/R	Decreases renal damage, serum creatinine and BUN. Reduces proinflammatory TNF-α and IL-6 levels. Attenuates NFκB signaling and increases p-JAK2 and p-STAT3 expression.	[209]
		Reduces serum and renal level of TNF-α, IL-1β, IL-12, IL-18 and INF-γ	[210]
		Decreases renal damage and serum creatinine. Decreases inflammatory chemokine expression, neutrophil infiltration, intracellular ROS production and cellular apoptosis. Reduces TLR4 and TNFα expression and inhibits NFκB and MAPK signaling pathways	[211]
	Rhabdomyolysis	Decreases serum creatinine levels, endothelial damage, inflammatory markers, redox balance and tubular cell death. Decreases TLR4 and Myd88 gene expression and activation of NFκB and ERK1/2 pathways	[45]
		Ameliorates renal damage, inflammation and apoptosis.	[212]
	Cisplatin induced AKI	Decreases serum urea and creatinine, reduces tubular necrosis, NFκB/p65 levels and caspase-3 expression	[213]
		Reduces renal histological injury, and plasma creatinine and BUN levels. Decreases renal MDA and restores renal GSH levels. Reduces ERK1/2 phosphorylation and NFκB expression. Decreases TNFα, IL-6, KIM-1 and NGAL mRNA expression.	[214]
	Doxorubicin induced AKI	Reduces proteinuria and podocyte injury. Ameliorates renal function, decreases oxidative stress and inhibits NFκB activation.	[215]
**Sulforaphane**	Cisplatin induced AKI	Reduces the activation of NFκB, p53, JNK and p38 pathways. Decreases TNF-α levels, expression of ICAM/VCAM and inflammatory infiltration	[216]
**Paclitaxel**	Sepsis induced AKI	Increases survival rate, downregulates TNF-α, IL-1β and IL-6 production, inhibits the expression and activation of NFκB.	[217]
**NaHS (H2S donor)**		Improves renal function and kidney histopathological changes, attenuates LPS-induced inflammation and oxidative stress, and reduces expression of TLR4, NLRP3, and caspase-1	[78]

## Data Availability

Not applicable.
